# Occurrence and characteristics of group 1 introns found at three different positions within the 28S ribosomal RNA gene of the dematiaceous *Phialophora verrucosa*: phylogenetic and secondary structural implications

**DOI:** 10.1186/1471-2180-11-94

**Published:** 2011-05-08

**Authors:** Kayoko Takizawa, Toko Hashizume, Katsuhiko Kamei

**Affiliations:** 1 Medical Mycology Research Center, Chiba University, 1-8-1 Inohana, Chuo-ku, Chiba, 260-8673, Japan; 2 Advanced Engineering Services Co. Ltd., Tsukuba Mitsui Building, 1-6-1 Takezono, Tsukuba, Ibaraki, 305-0032, Japan

## Abstract

**Background:**

Group 1 introns (ribozymes) are among the most ancient and have the broadest phylogenetic distribution among the known self-splicing ribozymes. Fungi are known to be rich in rDNA group 1 introns. In the present study, five sequences of the 28S ribosomal RNA gene (rDNA) regions of pathogenic dematiaceous *Phialophora verrucosa *were analyzed using PCR by site-specific primers and were found to have three insertions, termed intron-F, G and H, at three positions of the gene. We investigated the distribution of group 1 introns in this fungus by surveying 34 strains of *P. verrucosa *and seven strains of *Phialophora americana *as the allied species.

**Results:**

Intron-F's (inserted at L798 position) were found in 88% of *P. verrucosa *strains, while intron-G's (inserted at L1921) at 12% and intron-H's (inserted at L2563) at 18%. There was some correlation between intron distribution and geographic location. In addition, we confirmed that the three kinds of introns are group 1 introns from results of BLAST search, alignment analysis and Reverse Transcriptase-Polymerase Chain Reaction (RT-PCR). Prediction of secondary structures and phylogenetic analysis of intron sequences identified introns-F and G as belonging to subgroup IC1. In addition, intron-H was identified as IE.

**Conclusion:**

The three intron insertions and their insertion position in the 28S rDNA allowed the characterization of the clinical and environmental isolates of *P. verrucosa *and *P. americana *into five genotypes. All subgroups of introns-F and G and intron-H were characterized and observed for the first time in both species.

## Background

The type species *Phialophora verrucosa *was described by Medlar in 1915 [1] when he isolated the fungus from a human skin disease. The species is ubiquitous and cosmopolitan, and are important plant saprobes as well as human pathogens. Identification is based on conidial ontogeny and molecular systematics. Few studies involving molecular genotyping techniques have been reported for *P. verrucosa*. A study analyzed restriction fragment length polymorphisms (RFLP) of mitochondrial DNA to determine genetic variations and phylogenetic relationships among *P. verrucosa *strains [2].

Different molecular typing tools, such as random amplification of polymorphic DNA (RAPD), RFLP, pulsed-field gel electrophoresis (PFGE), multilocus enzyme electrophoresis (MLEE) and multilocus sequence typing (MLST), have been developed to provide a better understanding of the molecular epidemiology of fungal pathogens, e.g., *Candida albicans *[3-5] and *Aspergillus fumigatus *[6,7] and medically important filamentous fungi [8]. However, although the majority of the reported group 1 intron sequences have been found in a wide range of fungi (Comparative RNA Web [CRW] site: http://www.rna.ccbb.utexas.edu/[9], few studies about sequence and structure variation, distribution and phylogenetic relationships of introns from a single species have been performed in detail. We focused on group 1 introns within 28S rDNA from *P. verrucosa *to evaluate the prevalence of intron polymorphism at the strain level.

As the first step to determine intron sequence divergence, sequences of 28S rDNA of five representative strains of *P. verrucosa *were analyzed to find insertions. Based on these five sequences, site-specific primers were designed for use in PCR to detect insertions on other *P. verrucosa *and *P. americana *strains studied, in order to investigate incidence and distribution of insertions. Moreover, to characterize the insertions, we analyzed the phylogeny of the introns found in this study and predicted their secondary structures.

## Results

### Nucleotide structure of *P. verrucosa *28S sequences and the characterization intronic insertion

Since the sequence information of the 28S region was not available in public databases, we sequenced the 28S, including ITS, regions of five representative strains (Table 1) of *P. verrucosa*. Alignment of the five *P. verrucosa *rDNA sequences revealed the nucleotide sizes of 18S, ITS and 28S and the location of intron-F and G insertions (see Table 2 and Additional file 1). It was found that the sequences were composed of 29 nucleotides of partial 18S, 534-535 nucleotides of ITS and from 3349 to 4133 nucleotides of 28S regions. These genomic sequences were deposited at DDBJ and accession numbers are listed in Table 2. Twenty-five and two nucleotide substitutions were found within the ITS and D1D2 regions, respectively. The two bp substitutions in D1D2 were located at 1036 and 1042 nucleotide positions in the 28S region. These polymorphisms were confirmed in the five strains of *P. verrucosa *strains except for the two insertions in intron-F and G at 924 and 2239 positions of the 28S, respectively. None of the insertions were found in the Yao strain. BLAST comparisons showed with relatively high homology values that the sequences of both insertions were individually homogeneous to group 1 introns in the database. The positions of insertion for intron-F and G correspond to nucleotide positions 798 and 1921 of *Escherichia coli *23S nucleotide sequence accession number J01695 [10]. In addition, although these five strains of *P. verrucosa *did not possess intron-H, this region of PV28 strain was amplified with the site-specific primer pair (in Table 3) designed from the sequences obtained from another experiment (data not shown) and a 403-nucleotide insert representing intron-H was sequenced. The insertion positions of the intron-H correspond to nucleotide positions 2563 of *E. coli *23S nucleotide sequence.

**Table 1:** Thirty-four strains of *P. verrucosa *and seven strains of *P. americana *examined in this study


**Strain**	**Isolation source**	**Locality**		**Insertion**		**Genotype**	**Sample ID**
					
			**Intron-F**	**Intron-G**	**Intron-H**		

IFM 4928	Human	Japan	+	+	-	FG	PV1
IFM 5089	Human	Japan	+	-	-	F	PV2

IFM 51934	Human	China	-	-	-	N	Yao
IFM 41779	Human	China	+	+	-	FG	PV3
IFM 41750	Bark	China	+	-	-	F	TH9
IFM41710	Corn	China	+	-	na	F (na)	
IFM 41780	Human	China	+	-	-	F	
IFM 41721	Rotten wood	China	+	-	-	F	
IFM 41724	Rotten wood	China	+	-	-	F	
IFM 41725	Straw	China	+	-	-	F	
IFM 41739	Bark	China	+	-	-	F	
IFM 41740	Bark	China	+	-	-	F	
IFM 41746	Straw	China	+	-	-	F	
IFM 41749	Bark	China	+	-	-	F	
IFM 41752	Bark	China	+	-	-	F	
IFM 41764	Soil	China	+	-	-	F	
IFM 41755	Unknown	China	+	-	-	F	
IFM 41765	Soil	China	+	-	-	F	
IFM 41778	Straw	China	+	-	-	F	

IFM 41872	Soil	Colombia	-	-	-	N	
IFM 41871	Soil	Colombia	+	-	-	F	PV41
IFM 41879	Soil	Colombia	+	-	+	FH	
IFM 41881	Soil	Colombia	+	-	-	F	
IFM 41885	Soil	Colombia	+	-	-	F	

IFM 41884	Cactus	Venezuela	-	-	-	N	
IFM 41888	Rotten wood	Venezuela	-	-	-	N	
IFM 41887	Rotten wood	Venezuela	+	-	+	FH	PV28
IFM 41886	Cactus	Venezuela	+	-	+	FH	TH31
IFM 41892	Soil	Venezuela	+	-	+	FH	TH35
IFM 41883	Soil	Venezuela	+	-	-	F	
IFM 41893	Soil	Venezuela	+	-	-	F	

IFM 41897	Soil	Brazil	+	+	+	FGH	PV33
IFM 41898	Soil	Brazil	+	+	+	FGH	PV34
IFM 41899	Soil	Brazil	+	-	-	F	

*CBS 273.37	Human	Brazil	-	-	-	N	
*CBS 400.67	Soil	Brazil	-	-	-	N	
*CBS 281.35	Human	USA	-	-	-	N	
*CBS 220.97	Linden tree	USA	-	-	-	N	
*CBS 840.69	Decaying timber	Finland	-	-	-	N	
*CBS 221.97	Unknown	Uruguay	+	-	-	F	
*CBS 223.97	Human	USA	+	-	-	F	

*: *P. americana*, +: with insertion, -: no insertion, na: not analized.

**Table 2:** List of ITS, 28S rDNA and intron sequences of *P. verrucosa*


**Sample ID or entry name**	**Length (bp)**					**Splice positions**		**Accession number**
		
	**ITS**	**28S**	**Intron-F**	**Intron-G**	**Intron-H**	**position**^**a**^	**position**^**b**^	

PV1	535	4130						AB550775
PV2	535	3922						AB550776
PV3	535	4133						AB550777
PV41	534	3922						AB550778
Yao	535	3349						AB550779
F-PV1			391			924	798	
F-PV2			391			924	798	
F-PV3			391			924	798	
F-PV41			391			924	798	
G-PV1				390		2239	1921	
G-PV3				393		2239	1921	
F-TH9			389			924	798	AB550780
F-PV28			389			924	798	AB550781
F-TH31			389			924	798	AB550782
F-TH35			389			924	798	AB550783
F-PV33			390			924	798	AB550784
F-PV34			390			924	798	AB550785
G-PV33				389		2239	1921	AB550786
G-PV34				389		2239	1921	AB550787
H-PV28					403	2905	2563	AB611046

^a ^Position means relative to the 28S rRNA of *P. verrucosa *Yao strain and ^b ^position means relative to 23S rRNA of *E. coli *J01965.

**Table 3:** Primers used for the amplification and sequencing of *P. verrucosa*


**Primer**	**Sequence (5'-3')**	5' position*	**Source**	**5' position including ITS**

ITS1	TCCGTAGGTGAACCTGCGG	-563	White TJ, et al. [48]	1
ITS3	GCATCGATGAAGAACGCAGC	-309	White TJ, et al. [48]	255
NL1	GCATATCAATAAGCGGAGGAAA	39	O'Donnell K [49]	603
3PV26	CCGTCTTGAAACACGGACC	633	This work	1197
inFG-F	CCGAAAGATGGTGAACTATGCC	795	This work	1359
inF-F	ACGTGCAAATCGATCGTCAA	868	This work	1432
inF-R	CAAGGCCTCTAATCATTCGCT	1009	This work	1573
8PV26	GAACCTTTCCCCACTTCAG	1487	This work	2051
11PV26	AAGCCATAGGGAAGTTCCGT	1525	This work	2089
9PV26	GTCGTACTCATAACCGCAG	1818	This work	2382
CA-INT-L	ATAAGGGAAGTCGGCAAAATAGATCCGTAA	1881	McCullough MJ, et al. [50]	2445
2PV26	TCCCGAAGTTACGGATCTA	1918	This work	2482
16PV26	CCCAACCCTTAGAGCCAATC	1942	This work	2506
10PV26	CCGTACCAGTTCTAAGTTG	2089	This work	2653
inG-F	GATGGCCAGAAAGTGGTGTTG	2130	This work	2694
inG-R	TAGGGACAGTGGGAATCTCGT	2314	This work	2878
26S-INT3	CTAGCGAAACCACAGCCAAG	2323	This work	2887
CA-INT-R	CCTTGGCTGTGGTTTCGCTAGATAGTAGAT	2343	McCullough MJ, et al. [50]	2907
inFG-R	GCTCTCCCACCTATTCTACACC	2445	This work	3009
12PV26	TGGTATTTCACCGGCGATTG	2464	This work	3028
IGS1-M-R	CTGCCACAAGCCAGTTATC	2805	This work	3369
IGS3-M	CTTCGATGTCGGCTCTTCCT	2838	This work	3402
IGS-L	TAGTACGAGAGGAACCGT	2991	Williamson ECM et al. [51]	3555
3IGS-PV	TCTAAGTCAGAATCCGTGCCG	3090	This work	3654
5IGS1-PV	ACGAGCTACTGAGCGTAAG	3318	This work	3882
6IGS-PV	GACCACAGTCAGGCTTACG	3349	This work	3913
L2563 F	CACAGGGATAACTGGCTTGTGG	2781	This work	3345
L2563R	ATCTGAATCAACGGTTCCTCTCG	3018	This work	3582

* The 5' position is relative to the 28S rDNA sequence of the *P. verrucosa *Yao strain.

### Survey of insertions of *P. verrucosa *and *P. americana*

We amplified intron insertion regions using site-specific primer pairs we have designed for intron-F (inF-F and inF-R), intron-G (inG-F and inG-R) and intron-H (L2563F and L2563R), within the 28S region (Table 3). These primer pairs were used to screen and detect PCR amplicons for insertion regions within 34 *P. verrucosa *and seven *P. americana *strains. Amplicons were eluted in agarose gel to gain information regarding the intron insertions. No-insertion amplicons for intron-F and intron-G primers were in the size 142 and185 bps, respectively. When insertions were present, intron-F primer pair yielded amplicons in the size range from 531 to 533 bps, and intron-Gs in the size 575 or 578 bps. Moreover, amplicons of about 643 bps for intron-Hs were also eluted. It was revealed that there were 30 intron-F's, four intron-G's and six intron-H's within *P. verrucosa *and only two intron-Fs within *P. americana *as shown in Table 1. There was some correlation between intron distribution of *P. verrucosa *and geographic location, i.e., intron-Fs were found to have prevalence of 88% in *P. verrucosa *and intron-Hs were found specifically in the South American Continent. No introns were found except for two intron-Fs in *P. americana*. In addition, the agarose gel profiles allowed us to characterize genotypes and distribution frequencies of insertions from *P. verrucosa *including no-insertion as shown in Table 1. It was found that occurrence of genotypes F, FG, FH, FGH and N were at 64, 6, 12, 6 and 12%, respectively.

### Characterization of the *P. verrucosa *intronic insertion

**Figure 1:**
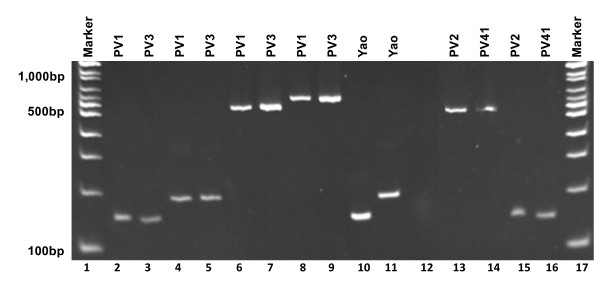
**Amplification pattern by RT-PCR with the site-specific primer pairs for intron-F and G**. PCR products of from cDNA amplified with the primers inF-F and inF-R are eluted in lanes 2, 3, 15 and 16, and with primers inG-F and inG-R in lanes 4 and 5. PCR products from genomic DNA amplified with primer pair for intron-F are eluted in lanes 6, 7, 10, 13 and 14, and with primer pair for intron-G in lanes 8, 9 and 11. Lane 12 is the negative control.

RT-PCR was carried out to identify the property of these insertions, namely, whether they are introns or unusual extensions incorporated into mature rRNA. Four representative strains were selected among the 41 strains surveyed. And it was found that two strains (PV1 and PV3) had two introns individually, while the other two strains (PV2 and PV41) had only one intron as shown in Figure 1. Insertions of strain PV1 and PV3 were eluted at 142 bps on lane 2 and 3 with intron-F primer pair, and 185 bps on lane 4 and 5 with intron-G primer pair, respectively. PV2 and PV41 exhibited 142 bps amplicons with intron-F primer pair as shown on lane 15 and 16, respectively. An intron-lacking Yao strain gave 142 and 192 bps amplicons with intron-F and G primer pairs on lane 10 and 11, respectively. The other lanes; namely, 6, 7, 8, 9, 13 and 14 show PCR products of genomic DNA as templates and lane 12 is negative control. These shows that all the insertions were excised after cDNA was transcribed, indicating that they were, as predicted, actively spliced introns. These results point to the possibility that these insertions are group 1 introns.

Moreover, we analyzed sequences of the spliced introns to confirm the boundaries of exon and intron sequences. The last nucleotide of the upstream exon was confirmed to be a T (U in RNA) and the last nucleotide of the intron was a G, consistent with group 1 introns [11,12].

### Phylogenetic relationships of introns F and G of *P. verrucosa*

Sequences of intron-F and G of ten *P. verrucosa *strains were sequenced and it was found that DNA sequence polymorphisms exist among the two introns, i.e., the intron-Fs ranged in the size from 389 to 391 bps and the four intron-Gs from 389 to 393 bps shown in Table 2. There were 24 nucleotide substitutions and two deletions/insertions (TH9 strain) within intron-F. There were five nucleotide substitutions among intron-Gs from PV1, PV33 and PV34, unlike 36 substitutions between PV1 and PV3. In addition, Blast search analyses and alignment lead us to believe that intron-Fs and Gs from 14 introns belong to subgroup IC1 of group 1 intron.

**Figure 2:**
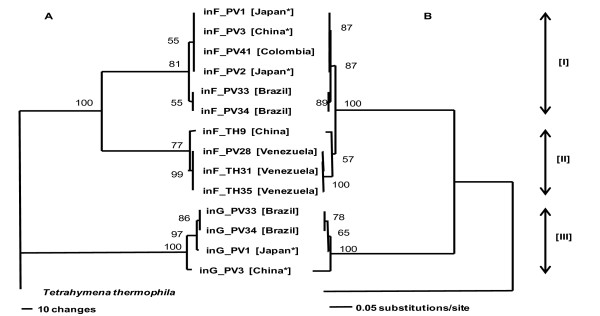
**Phylogenetic relationship of intron-F and G within 28S of *P. verrucosa***. The trees were generated using MP (A) and NJ (B). One of three equally MP trees (tree length = 353, consistency index (CI) = 0.9575, homoplasy index (HI) = 0.0425, CI excluding uninformative characters = 0.9268, HI uninformative characters = 0.0732, retention index = 0.9679, rescaled consistency index = 0.9268). * indicates a clinical isolate of *P. verrucosa*.

Fourteen introns from 12 representative strains of *P. verrucosa *including *Tetrahymena thermophila *as out-group were aligned and used for phylogenetic analyses. Neighbor-joining (NJ) and Maximum Parsimony (MP) trees based on the alignment of these intron sequences are shown in Figure 2. The data set consisted of 466 characters, of which 156 were removed from the MP analysis due to ambiguous alignment. Of the remaining 310 characters, 201 were variable and 129 were phylogenetically informative for parsimony analysis. Three major distinct and well-supported clades that had homologous topology were obtained from both phylogenetic analysis methods showing that all the introns analyzed were undergoing a similar rate of evolution. The first clade [I] (87% BS support in NJ, 81% in MP) consisted of six strains having intron-F including 3 clinical isolates, the second clade [II] (57% BS in NJ and 77% in MP) consisted of 4 strains having intron-F, and the third clade [III] (100% BS in both trees) consisted of four G introns. All the introns clustered in clades [I] and [II] are inserted at the same position L798 those in clade [III] at the same position L1921. Introns inserted at the same positions belong to the same clusters and are considered to be the same subgroups. We observed that two Venezuelan strains in clade [II] (TH31 and TH35) included the remnants of homing endonuclease (HE) gene sequences, which were found to invade into IC1 introns of the 18S regions in another study (unpublished data).

### Alignment and phylogenetic analysis of the core regions of the group IC1 introns

**Figure 3:**
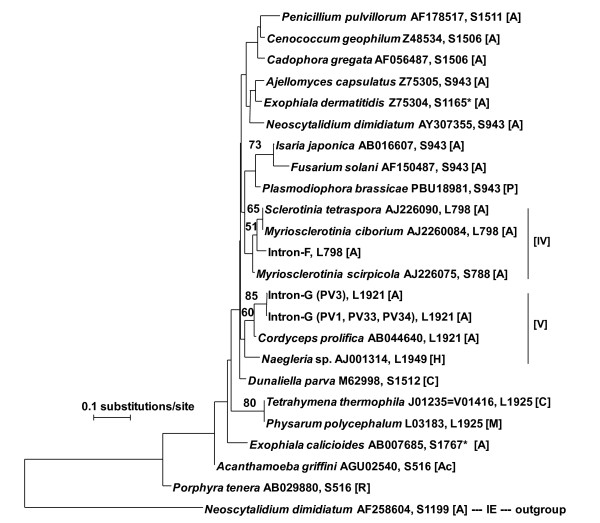
**Phylogenetic tree of IC1 intron based on elements P, Q, R, S and a segment of P3**. Numerals at each node are bootstrap probabilities from NJ analysis. Insertion positions are given after the sample ID or accession number. * indicates the insertion position relative to the 18S rDNA of the *S. cerevisiae *sequence.

Alignment of the core regions consisting of highly conserved sequences of the elements of P, Q, R and S and the pairing segment P3 and the nucleotide sequences, in particular, the last two nucleotides GC of the Q element and the first and second GU nucleotides of the R element [12] (Additional file 2) showed that the introns belong to group IC1. All core region sequences of intron-Fs were found to be identical. Two sequences of core regions termed as intron-G (PV3) and intron-G (PV1, PV33, PV34) were obtained and added to the NJ analysis in Figure 3. The NJ tree was constructed based on the alignment of these core regions consisting of three representative sequences of *P. verrucosa *and IC1 of 21 taxa drawn from database using IE intron from *Neoscytalidium dimidiatum *as out-group. The phylogeny of intron-F and G formed separate clades as shown in Figure 3, and indicated that both introns were likely acquired independently. Indeed, all intron-Fs were found to be closely related to *Myriosclerotinia ciborium *and *Sclerotinia tetraspora *introns which are located at L798. Two sequences of intron-G located at L1921 were grouped together with 85% BS value and found to be on the neighboring clade with *Cordyceps prolifica *intron located at L1921. The phylogenetic tree suggests that both introns may be inserted prior to the divergence of the species formerly belonging to clade [IV] and [V]. Collectively, this tree displays that all introns of *P. verrucosa *generated by the core regions are members of subgroup IC1s.

### Modeling of the *P. verrucosa *insertions revealed that they were group IC1 introns

**Figure 4:**
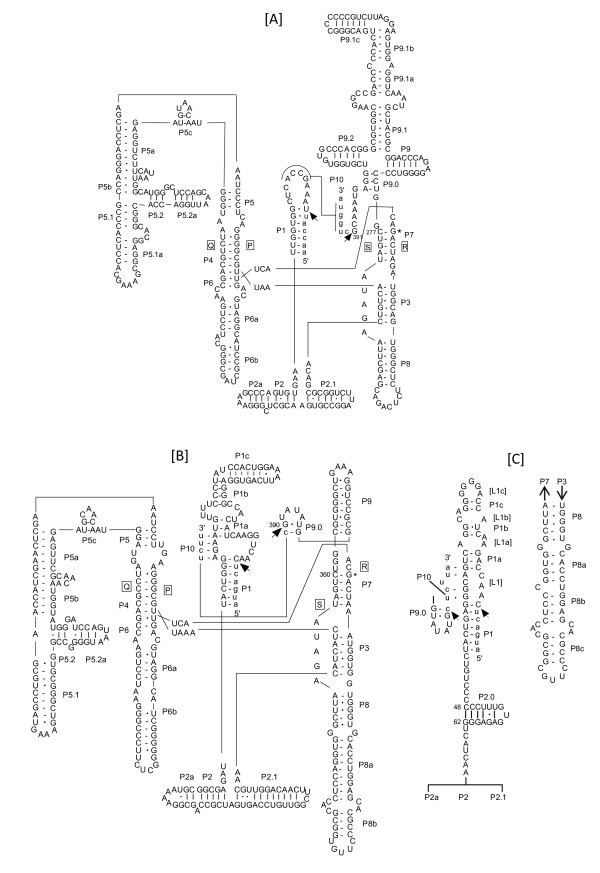
**A-C. - Diagrams for predicted secondary structure of *P. verrucosa***. **[A]**: intron-F from rDNA of PV1, **[B]**: intron-G from PV1 and **[C]**: intron-G from PV3. Capital letters indicate intron sequences and lowercase letters indicate flanking exon sequences. Arrows point to the 5' and 3' splice sites. The guanosin cofactor-binding sites are marked with *.

The predicted secondary structure of the intron-F and G were constructed as follows. The conserved P, Q, R and S regions of intron-F (L798) from PV1 were initially aligned with the same regions from other taxa, and then regions of P1 through P10 were constructed and added on the basis of the secondary structure model as shown in Figure 4[A][13]. The P10 region was formed using the internal guide sequence (IGS). The catalytic core was defined by a set of structurally conserved elements, including elements P3 to P8. A G-C pair within P7, i.e. G391-C277 of intron-F was assumed to be G-binding positions [14]. Extended P5 and P9 stems were displayed in the putative structure of intron-F from PV1. Nine intron-Fs from nine strains (PV2, 3, 28, 33, 34 and 41 and TH9, 31 and 35) of *P. verrucosa *were predicted to be the same structures as the putative structure of intron-F derived from PV1 drawn in Figure 4[A], alternatively, shown in Additional file 3. These nucleotide variations among intron-F were observed mainly in the loop and at four positions where one nucleotide of P5a, two of P5.1a and one of P5.2 stem were positioned. The base pairs GU and CG within P6 were formed in the core region of intron-F [12]. The nucleotides A71, A72, U73 were located in segments J3/4 of PV1 intron-F [15-18]. These predictions of secondary structure revealed that all intron-Fs were IC1 group 1 introns.

The structure of intron-G (L1921) from PV1 was drawn just as was done for intron-Fs (Figure 4[B]). A G-C pair within P7, i.e. G390-C360, was assumed to be the G-binding positions. The GU-CG pair of P6 and the AAU in J3/4 was the same as in the intron-F core region of PV1. This putative intron-G exhibited expanded regions of P1 and P5. The three intron-Gs of PV1, PV33 and PV34 were found to be similar among the three strains. Different features were found in PV3 as shown in Figure 4[C] wherein the sequence of PV3 differed in P1 region among four trains; namely, short stems in P1b and P1c and small bulge loops of L1 and L1a (Additional file 4). Moreover, PV3 added P2.0 and P8c, although the other intron-Gs did not. Prediction structures in the remaining two introns of PV33 and PV34 are not shown. Nevertheless, all subgroups of intron-G were also identified as IC1, based on comparison of tertiary structures across segments P3-7 of the four strains.

In conclusion, we have identified that the ten intron-Fs and four intron-Gs of *P. verrucosa *belong to IC1 group 1 introns.

### Characterization of intron-H

**Figure 5:**
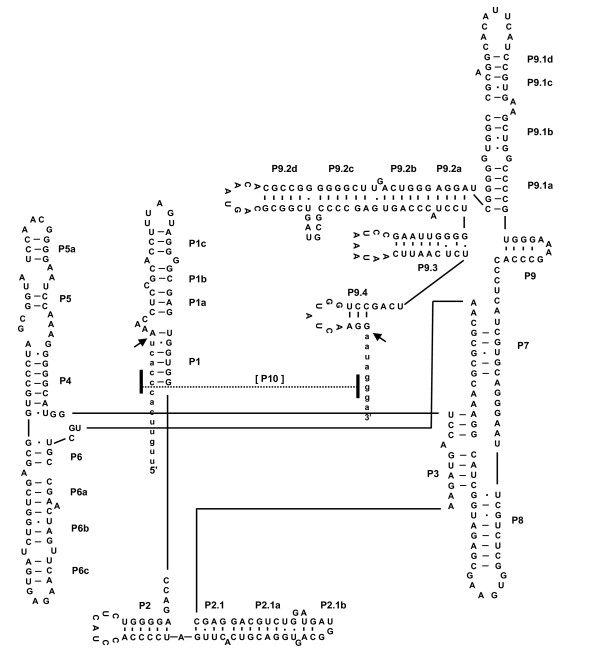
**Diagrams for predicted secondary structure of intron-H from PV28 strain**. Capital letters indicate intron sequences and lowercase letters indicate flanking exon sequences. Arrows point to the 5' and 3' splice sites.

Loss of P5abcd domain in derived S788 introns was correlated with inability to self-splice *in vitro *in a previous report [19]. Accordingly, we have not confirmed insertion positions of intron-H by RT-PCR. However, we examined PV-28 strain as the representative strain of intron-H by analyzing the sequence alignment of the core region of subgroup IE from other organisms in the database. Moreover, we predicted the secondary structure of this intron-H as shown in Figure 5. The secondary structure modeling revealed distinctive IE sequences, namely, the non-expanding P5 and the expanding P9 regions.

## Discussion

To date, although a variety of introns from eukaryotes have been described in the rRNA gene loci of fungi [9], few introns in *Phialophora *species have been reported. An unusually small group 1 intron of 67 bps from the nuclear 18S rDNA has been described in a splicing study of *Capronia semiimmersa*, a teleomorph of *P. americana *which is known to be similar to *P. verrucosa *[20-22]. These small introns contain only P1, P7 and P10 elements, because most of the core regions common in almost all other group 1 introns are missing. Four intron sequences have been reported or registered in dematiaceous fungi; namely, 283 bps within the small subunit (SSU) rDNA from *Cadophora gregata *f. sp. *adzukicola *[23], 339 bps within SSU from *Cadophora finlandica *(accession number: AF486119), 456 bps within the large subunit (LSU) rDNA from *C. semiimmersa *[24] and 397 bps within LSU from *Cladophialophora carrionii *[24]. These introns have not been subjected to secondary structure analysis. Therefore, we aimed to identify the introns that we found in this study and to investigate the prevalence and phylogenetic relationships of 28S group 1 intron at the intra-species level.

The intron-F, G and H in the 28S rDNA of both species were found to belong to two subgroups, IC1 and IE, of group 1 intron. IC1 at L798 is the most common insertion position as shown in Table 1 and in the CRW website, and insertions at L1921 and L2563 were found comparatively in the database. The loss of most of P5 in the secondary structure of intron-H is believed to be a relatively recent evolutionary event [19]. The three insertions possessed all the ten elements (P1-P10) common in group 1 introns. Enzymatic core regions are especially well conserved in primary and secondary structures, as described in previous reports [12,25], suggesting that they were derived from a common origin. Peripheral elements of the core have various forms and these variations have been used to subdivide introns into five major subgroups [17,26]. In this study, the phylogeny obtained in Figure 2 and 3 showed that all IC1 introns inserted into *P. verrucosa *have been surviving with base substitution/insertion/deletion, especially among peripheral elements as a consequence of some events after the individual insertion of IC1 at L798 and L1921, and may have spread by homing (e.g., [27-29]) or reverse splicing [30-32].

Comparisons of intron-F and G indicate comparative high sequence divergence within *P. verrucosa *wherein the sequence similarity among intron-F's was 94%, and 99% among intron-G's with the exception of PV3 and 90% among all the four intron-G's. The phylogenetic relationships among both introns were very similar to each other (Figure 2). These results suggest that they have diverged from a common origin.

Thirty isolates of *P. verrucosa *and two isolates of *P. americana *possessed intron-F, G and H either individually or as a combination of these introns. Genotypes based on the combinations of presence or absence of introns, type and position of insertions were established to discriminate among the isolates surveyed. As a result, five genotypes; namely, F, FG, FH, FGH and N were identified, as shown in Table 1. Type-F was isolated in all the countries where the strains used in the study. Intron distribution was found to have some correlation with geographic location, albeit the number of isolates used was small. For example, most of the Chinese isolates except for Yao-strain had only type-F. Isolates from South American continent had slight tendency to have an intron-H, wherein they were either type-FH or FGH. Intron-G's occurred as type-FG in the clinical isolates of Japan and China and as two type-FGH's in soil isolates of Brazil. In addition, according to an interpretation from a different viewpoint, insight into possible correlation of geographic origin among introns from *P. verrucosa *strains have emerged from these insertion position results, namely, the spread of L798 among a large number of *P. verrucosa *isolates and the existence of L1921 and L2563 that coexist with the other intron insertions among the species and strains that have lost introns. L1921 positions are only seen in two clinical isolates from Japan and China and two isolates from Brazilian soil. The L2563 position seems to be specific to the South American continent in six environmental isolates. The possible correlation tendencies shown in our results have also been reported in previous studies described below. For example, group 1 intron *Cg*SSU was found in the SSU rDNA of deuteromycetes mycorrhizal fungus *Cenococcum geophilum*, and the intron-positive isolates occurred mainly in North America and Europe and negative isolates in Western, Midwestern and Southern North America [33]. In other studies on intron distribution from a single species, four different group 1 intron combinations within LSU rDNA from entomopathogenic hyphomycete *Beauveria bassiana *were divided into 13 genotypes to investigate distribution frequencies in the population and it was found that there was a tenuous correlation with geographic origin or insect host species [34]. Moreover, M. Márquez, et al. have found three intron insertion positions within LSU rDNA and established seven genotypes among 26 biocontrol isolates for entomopathogenic anamorphic *Metarhizum anisopliae *[35].

Meanwhile, we found that five isolates of *P. americana *had no introns, even though two isolates were detected as type-F. Intron-loss strains might have been lost from taxa possessing intron-F in the common ancestor of the species during its evolution [36]. Further, the lateral transfers appear to have been rare events in *P. americana*. The fact that intron-F was found in almost all isolates of *P. verrucosa*, it is believed that intron-F may be specific to *P. verrucosa*. To confirm this hypothesis, more isolates are needed in the survey and the relationships of the clinical background of the individual patients and the ecological niches of saprobic isolates must be investigated. Further analysis of genotypes within the complete nuclear rDNA gene must be done and the presence of HE gene sequences must be analyzed since they provide key information on intron phylogeny and origin. This study is a first step in the study of introns in *P. verrucosa *and *P. americana*.

## Conclusion

The three insertions within 28S rDNA of clinical and environmental isolates of *P. verrucosa *and *P. americana *allowed us to characterize them into five genotypes using agarose gel electrophoresis patterns. The two insertions, namely, intron-F and G, were characterized as subgroup IC1 by subjecting them to RT-PCR, secondary structure and phylogenetic analysis to determine whether they are true introns, to characterize subgroup and to infer evolutionary relationships, respectively. Another insertion, intron-H, was characterized as an IE intron using BLAST search and by prediction of secondary structure. Furthermore, we also developed a system to classify genotypes based on the presence and distribution of group 1 introns and the distributions as DNA polymorphism among the two species.

## Methods

### Fungal strains and culture conditions

We studied 34 *P. verrucosa *strains including of five clinical isolates as shown in Table 1. Seven *P. americana *strains including of three clinical isolates were used as allied species. All the isolates were preserved by using L-drying method and were sub-cultured on potato dextrose ager (Difco) slant before extraction of genomic DNA. For an extraction of total RNA, liquid cultivation was performed in 50-ml Erlenmyer flask containing 20 ml of potato dextrose medium at 30°C for seven days on a rotary shaker at 120 rpm.

### Extraction of genomic DNA and total RNA

DNA extraction was performed using an InstaGene Matrix extraction kit (BioRad, Hercules, CA, USA) according to the manufacturer's instructions with minor revisions. Particularly, cells were ground with micro pestle before incubation at 56°C. The extracted DNA was then diluted 1:10 and used as template DNA for PCR amplification.

Total RNA was extracted by using the Nucleic Acid Purification Kit MagExtractor (TM -RNA- TOYOBO, Osaka, Japan). The following procedures were done before carrying out the manufacturer's instructions. Approximately 20 mg (wet weight) of mycelia were washed with water and then rinsed with *Schizosaccharomyces pombe *spheroplast buffer (20 mM citrate-phosphate buffer (pH 5.6), 50 mM EDTA and 0.9 M sorbitol). This was followed by addition of 100 μl of buffer plus 20 units of Lyticase (L-5263; SIGMA, MO, USA) and 0.01 units of Chitinase (C-7809; SIGMA, MO, USA). The suspension was mixed by vortexing, incubated at 37°C for 30 min and added with 700 μl of a lysis-adsorption solution including 1% 2-mercaptoethanol and pricked with a pipette tip until the solution became viscous. The extracted RNA was treated with RNase-Free DNase Set (QIAGEN). Approximately more than 20 ng/μl RNA was obtained.

### PCR amplification and sequencing analysis

A primer walking method was performed to obtain the sequences of the entire 28S rDNA region including ITS. PCR Master Mix (Promega, Madison, WI, USA) and TaKaRa LA Taq (TAKARA Bio Inc, Sigma, Japan) were used depending on the amplification sizes. The PCR conditions for PCR Master Mix consisted of denaturation for 4 min at 95°C, followed by 30 amplification cycles of denaturation at 94°C for 1 min, annealing at primer-dependent temperatures based on Tm values for 1 min and extension at 72°C for 1.5 min, and then 1 cycle of 5 min at 72°C. For TaKaRa LA *Taq *consisted of denaturation for 1 min at 94°C, followed by 30 cycles of denaturation at 98°C for 5 sec, annealing at primer-dependent temperatures for 30 sec and extension at 72°C for 2 min, and then 1 cycle of 72°C for 10 min. PCR products were purified with SAP-IT (USB Corporation, Cleveland, OH, USA) and then sequenced with primers listed in Table 2 and the BigDye Terminator v3 Cycle Sequencing Kit (Applied Biosystems, Foster City, CA, USA) on an ABI Prism 3130 × l Sequencer (Applied Biosystems, Hitachi). The nucleotide sequences were determined from both strands. To determine base substitutions and intron insertion positions, sequences were aligned by using the alignment function of GENETYX ver. 9.1.1 (GENETYX COOPERATION, Tokyo, Japan).

### Determining incidence of introns by agarose gel

The extracted DNA was used as template DNA for the amplification of the insertion regions (intron-F, G and H). PCR was performed individually using PCR Master Mix and the primer pair inF-F and inF-R for intron-F and inG-F and inG-R for intron-G which we newly designed. Primer pair L2563F and L2563R for intron-H was designed based on sequences of exon and group 1 intron on CRW website, because the intron was not inserted in the five representative strains used. PCR conditions were the same as described above and the resulting DNA fragments were resolved by electrophoresis on a 2% agarose gel (NuSieve^® ^3:1 Agarose, TAKARA Bio Inc, Sigma, Japan) in Tris-borate-EDTA buffer. Presence or absence of individual intron was listed as positive/negative in Table 1. In addition, the strains were categorized into five intron types; namely, F, FG, FH, FGH and N on the basis of the intron insertions.

### RT-PCR and colony sequencing

The RT-PCR from total RNA was performed using a SuperScript ™ III One Step RT-PCR System with Platinum *Taq *DNA Polymerase (Invitrogen, CA, USA) according to the manufacturer's instructions. Ten microliters of RNA was used for a 50 μl reaction mixture consisting of 25 μl of ReactionMix, 2 μl of SuperScript™ III RT/Platinum *Taq *Mix, 1 ul of each primer inF-F (10 μM) and inF-R (10 μM) for RT-PCR of intron-F and 11 μl of autoclaved distilled water. For RT-PCR of intron-G, primer pair inG-F and inG-R was used. RT-PCR was carried out in the following conditions: cDNA synthesis at 55°C for 30 min, denaturation at 94°C for 2 min, and PCR amplification at 40 cycles of 94°C for 15 sec, 55°C for 30 sec and 68°C for 1.5 min and final extension at 68°C for 5 min. Amplification products were eluted in 3.5% polyacrylamide gel in tris-acetate-EDTA buffer on an electrophoresis run condition of 100 V for 30 min and followed by 75 V for 25 min, together with genomic DNAs amplified with the same primer pairs as control (shown in Figure 1).

The RT-PCR products were purified with the SUPREC-PCR (TAKARA Bio Inc, Sigma, Japan) and ligated into the pGEM-T Easy Vector System (Promega, Madison, WI, USA). Plasmids were transformed into *E. coli *competent cells (ECOS TM Competent *E. coli*, JM109, NIPPON GENE Co., LTD., Japan). Transconjugants were selected on LB agar plates containing 50 μg/ml ampicilin and 40 μg/ml of 5-bromo-4-chloro-3-indoyl-β-D-galactopyranoside (X-Gal). The presence of the expected insert was confirmed by PCR and agarose gel electrophoresis. The inserts were sequenced with T7 (5'-TAATACGACTCACTATAGGG-3') and M13 reverse primers (5'-AGGAAACAGCTATGACCATGA-3').

### Phylogenetic analysis of introns from *P. verrucosa*

Nucleotide sequences were aligned using the BioEdit program version 7.0.9.0 [37]. For phylogenetic analysis, alignment gaps were treated as missing data and ambiguous positions were excluded from the analysis. NJ analysis [38] as distance matrix method and MP analysis as character state method were carried out using PAUP 4.0b10 [39]. For NJ analysis, the distances between sequences were calculated using Kimura's two-parameter model [40]. MP analysis was undertaken with the heuristic search option using the tree-bisection-reconstruction (TBR) algorithm with 1000 random sequence additions to find the global optimum tree. All positions were treated as unordered and unweighted. The maximum tree number was set at 10^4^. To estimate clade support, the bootstrap procedure of Felsenstein [41] was employed with 1000 replicates in both MP and NJ analyses. Bootstrap (BS) values higher than 50% are indicated.

### Alignment and phylogenetic analysis of core sequences

For the comparison with highly conserved sequences of subgroup IC1 from 20 taxa, sequences of elements of P, Q, R and S and the pairing segment P3 were obtained from DDBJ database (accession numbers shown after sample name in Figure 3). These regions do not include IGS, because the sequences in the upstream region of intron insertion positions do not share a common IGS [42]. The NJ tree was constructed after alignment of all the sequences, which ranged from 57 to 60 bps (Additional File 2). Insertion positions are shown after the sample ID or accession number. The insertion position numbering of the taxa refers to the 23S nucleotide sequence of *E. coli *[10] except for the *Exophiala calicioides *which is based on the 18S of *Saccharomyces cerevisiae*. Taxonomic affiliation was indicated by letters in parentheses; namely, [A], Fungi/Ascomycota; [Ac], Acanthamoebidae; [C], Chlorophyta; [H], Heterolobosea; [M], Mycetozoa and [R], Rhodophyta.

### Secondary structure modeling

The secondary structures are proposed from modeling by Michel et al. [14,26,43] and computational analysis was done using the Mfold web server available at http://mfold.rna.albany.edu/[44] and GENETYX Ver.9 software, with manual adjustments. The pairing segments of P1-P10 locations are indicated in Figure 4 and 5. Moreover, the model was manually optimized based on previous studies of group 1 introns [17,45-47].

## Authors' contributions

KT: conceived the study, designed the experimental plan, performed the experiments, wrote and revised the manuscript. TH: performed the experiments. KK: participated in the coordination of the study, helped draft and revise the manuscript. All authors read and approved the final manuscript.

## Supplementary Material

Additional file 1 **Schematic representation of the large ribosomal subunit 28S gene**. The *hatched *and *dotted *boxes correspond to the group 1 intron of *P. verrucosa *inserted at positions 798, 1921 and 2563 relative to the 23S rDNA of the *E. coli *J01965 sequence. The numbering in the parentheses is relative to the ITS and 28S rDNA sequence of *P. verrucosa*. Click here for file

Additional file 2 **Partial alignment of IC1 introns of *P. verrucosa *and selected introns from the database**. Highly conserved sequences of the elements of P, Q, R and S and the pairing segment P3 are also shown. Intron insertion positions relative to *E. coli *are given after the sample ID or taxon name. * indicates the insertion position relative to the 18S rDNA of the *S. cerevisiae *sequence. Letters in parentheses indicate taxonomic affiliation: [A], Fungi/Ascomycota; [Ac], Acanthamoebidae; [C], Chlorophyta; [H], Heterolobosea; [M], Mycetozoa; [R], Rhodophyta. Click here for file

Additional file 3 **Alignment of intron-F used for the phylogenetic analysis and the modeling of secondary structure**. The gaps were marked with *dashes*. The highly conserved (ribozymatic core) regions of the P, Q, R and S were marked with *dotted line*s. *Boxed *nucleotides participate in the pairing segments of P1-P10 of the secondary structure model. Click here for file

Additional file 4 **Alignment of intron-G used for the phylogenetic analysis and the modeling of secondary structure**. The gaps were marked with *dashes*. The highly conserved (ribozymatic core) regions of the P, Q, R and S were marked with *dotted line*s. *Boxed *nucleotides participate in the pairing segments of P1-P10 of the secondary structure model. Click here for file
