# Supplementary figures and images for: Occurrence and characteristics of group 1 introns found at three different positions within the 28S ribosomal RNA gene of the dematiaceous Phialophora verrucosa: phylogenetic and secondary structural implications
Source: BMC Microbiol. 2011 May 8;11:94. doi: 10.1186/1471-2180-11-94 (PMC3112068; doi:10.1186/1471-2180-11-94)

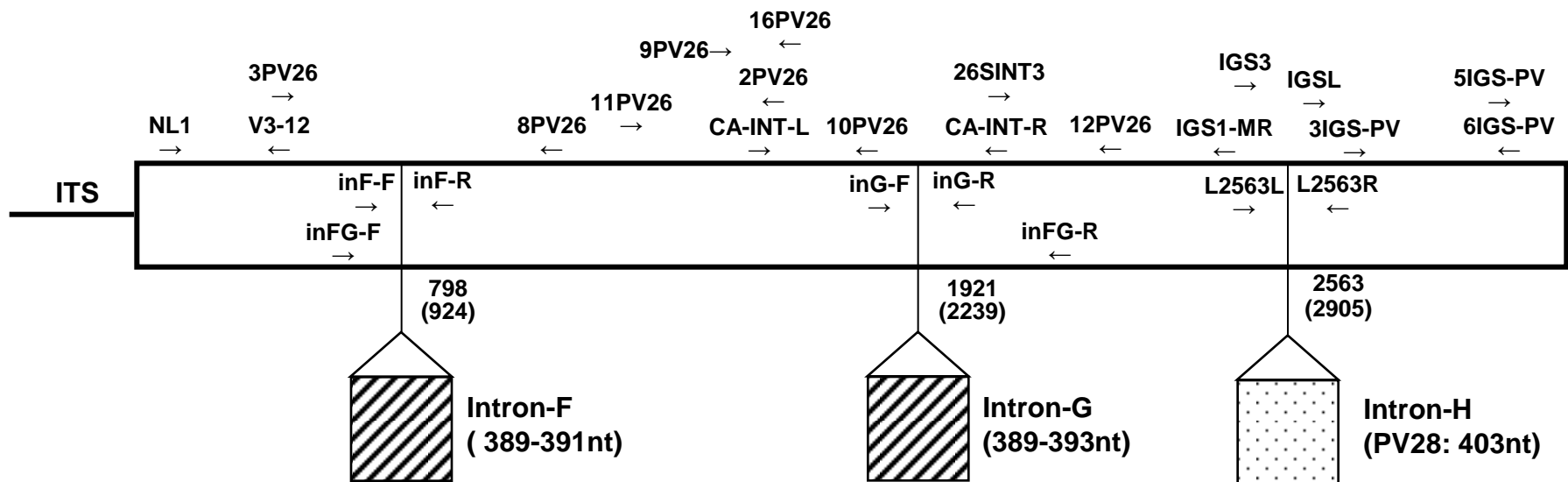

Supplement: Additional file 1 — Schematic representation of the large ribosomal subunit 28S gene. The hatched and dotted boxes correspond to the group 1 intron of P. verrucosa inserted at positions 798, 1921 and 2563 relative to the 23S rDNA of the E. coli J01965 sequence. The numbering in the parentheses is relative to the ITS and 28S rDNA sequence of P. verrucosa. [file 1471-2180-11-94-S1.PDF]
