# Supplementary material for: Occurrence and characteristics of group 1 introns found at three different positions within the 28S ribosomal RNA gene of the dematiaceous Phialophora verrucosa: phylogenetic and secondary structural implications
Source: BMC Microbiol. 2011 May 8;11:94. doi: 10.1186/1471-2180-11-94 (PMC3112068; doi:10.1186/1471-2180-11-94)
Supplement: Additional file 2 — Partial alignment of IC1 introns of P. verrucosa and selected introns from the database. Highly conserved sequences of the elements of P, Q, R and S and the pairing segment P3 are also shown. Intron insertion positions relative to E. coli are given after the sample ID or taxon name. * indicates the insertion position relative to the 18S rDNA of the S. cerevisiae sequence. Letters in parentheses indicate taxonomic affiliation: [A], Fungi/Ascomycota; [Ac], Acanthamoebidae; [C], Chlorophyta; [H], Heterolobosea; [M], Mycetozoa; [R], Rhodophyta. [file 1471-2180-11-94-S2.PDF]

	P3	P	Q	R	S	
inF-PV_all-L798[A]	CGACACUG-UCA	AAUUG--C-GGGGAC	AAUCUGCAGC	GUUCACAGACUAG	AAGAUAUAGUCG	58bp
inG-PV1_33_34-L1921[A]	CAACAUCA-UCA	AAUUG--C-GGGAAG	GAUCCGCAGC	GUUCAACGACUAA	AAGAUAUAGUCU	58bp
inG-PV3-L1921[A]	CAACAUCA-UCA	AAUUG--C-GGGAAG	AAUCCGCAGC	GUUCAACGACUAA	AAGAUAUAGUCU	58bp
A. capsulatus-S943[A]	CGACACAC-UCA	AACUG--C-GGGAAA	AAUCCGCAGC	GUUCAGAGACUAA	AAGAUAUAGUCC	58bp
A. griffini-S516[Ac]	----UGUACACC	GUAAAUUGCGGGAAA	AAUCCGCAGC	GUUCAGAGACUAU	AAGGUAUAGUC-	57bp
C. geophilum-S1506[A]	CGACACUU-UCG	AAUUG-AC-GGGGAU	AAUCCGCAGC	GUUCACAGACUAA	AAGAUAUAGUCG	59bp
C. gregata-S1506[A]	CAACACGA-UCG	AAUUG--C-GGGGAC	GAUCCGCAGC	GUUCACAGACUAA	AAGAUAUAGUCG	58bp
C. prolifica-L1921[A]	CGACACCG-UCA	AAUUG--C-GGGAAG	GAUCCGCAGC	GUUCAACGACUAA	AAGAUAUAGUCU	58bp
D. parva-S1512[C]	-AACACUGAUCA	AAUUG--C-GGGAA-	AAUCCGCAGC	GUUCAGAGACUAA	AAGAUAUAGUCC	57bp
*E. calicioides*_S1767* [A]	CAACACCG-UCA	AAUUG--C-GGGAAC	AAUCCGCAGC	GUUCACAGGCCAA	AAGAUAUGGUCG	58bp
*E. dermatitidis*_S1165* [A]	----CACCCUCA	AACUGUUCGGGGAAG	GAUCCGCAGC	GUUCAGAGACUAA	AAGAUAUAGUCC	58bp
F. solani-S943[A]	CGACAUCC-UCA	AAUUG--C-GGGAAA	GAUCCGCAGC	GUCCAGAGACUUG	AAGAUAAAGUCC	58bp
I. japonica-S943[A]	CGACACCC-UCA	AAUUG--C-GGGAAA	GAUCCGCAGC	GUUCAGAGACUUG	AAGAUAAAGUCC	58bp
M. ciborium-L798[A]	CGACAUUG-UCA	AAUUGUUC-GGGGAC	UAUCCGCAGC	GUUCACAGACUAG	AAGAUAUAGUCG	60bp
M. scirpicola-S788[A]	CAACACCG-UCA	AAUUG--C-GGGGAC	AAUCCGCAGC	GUUCACAGACUAG	AAGAUAUAGUCG	58bp
Naegleria sp.-L1949[H]	CGACACUG-UCA	AAUUG--C-GGGGAG	AAUCCGCAGC	GUUCAUCGACUAA	AAGAUAUAGUCA	58bp
N. dimidiatum-S943[A]	CGACACGC-CCA	AAUUG--C-GGGGAG	AAUCCGCAGC	GUUCAGAGACUAA	AAGAUAUAGUCC	58bp
P. brassicae-S943[P]	CGACAUCG-UCA	AAUUG--C-GGGAAC	GAUCCGCAGC	GUUCAGAGACUAG	AAGAUAUAGUCC	58bp
P. polycephalum-L1925[M]	CGAGACCG-UCA	AAUUG--C-GGGAAA	ACCACGCAGC	GUUCACAGACUAA	AAGAUAUAGUCG	58bp
P. tenera-S516[R]	----AUACACUU	CUAAAUUGCCGGGGA	GAUCCGCAGG	CUUCAGAGACUCU	AAGGGAGAGUCC	58bp
P. pulvillorum-S1511[A]	CGACACUC-UCG	AACUG-CC-GGGGA-	AAUCCGCAGC	GUCCACAGACUAA	AAGAUAUAGUCG	58bp
S. tetraspora-L798[A]	CGACAUUG-UCA	AAUUGUUC-GGGGAC	UAUCCGCAGC	GUUCACAGACUAG	AAGAUAUAGUCG	60bp
T. thermophila-L1925[C]	CAAGACCG-UCA	AAUUG--C-GGGAAA	ACCACGCAGC	GUUCACAGACUAA	AAGAUAUAGUCG	58bp
	....	....**..	.....****.	. *.** .*.*...	***..*..***	
