# Supplementary material for: Occurrence and characteristics of group 1 introns found at three different positions within the 28S ribosomal RNA gene of the dematiaceous Phialophora verrucosa: phylogenetic and secondary structural implications
Source: BMC Microbiol. 2011 May 8;11:94. doi: 10.1186/1471-2180-11-94 (PMC3112068; doi:10.1186/1471-2180-11-94)
Supplement: Additional file 3 — Alignment of intron-F used for the phylogenetic analysis and the modeling of secondary structure. The gaps were marked with dashes. The highly conserved (ribozymatic core) regions of the P, Q, R and S were marked with dotted lines. Boxed nucleotides participate in the pairing segments of P1-P10 of the secondary structure model. [file 1471-2180-11-94-S3.PDF]

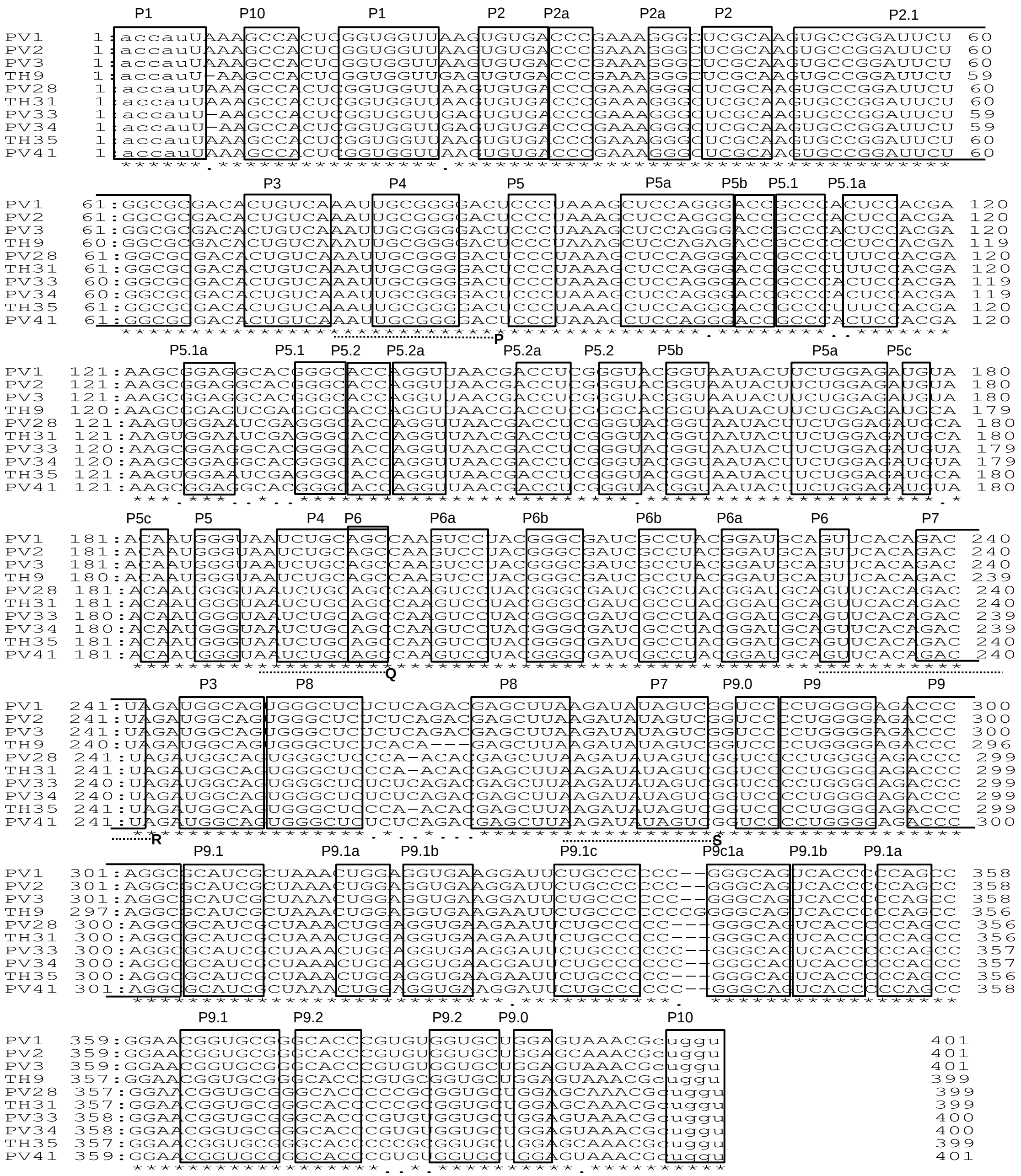
P1 P10 P1 P2 P2a P2a P2 P2.1
PV1 1:accauUAAAGCCACUCGGUGGUUAAGUGUGACCCGAAAGGGCUCGCAAGUGCCGGAUUCU 60
PV2 1:accauUAAAGCCACUCGGUGGUUAAGUGUGACCCGAAAGGGCUCGCAAGUGCCGGAUUCU 60
PV3 1:accauUAAAGCCACUCGGUGGUUAAGUGUGACCCGAAAGGGCUCGCAAGUGCCGGAUUCU 60
TH9 1:accauU-AAGCCACUCGGUGGUUGAGUGUGACCCGAAAGGGCUCGCAAGUGCCGGAUUCU 59
PV28 1:accauUAAAGCCACUCGGUGGUUAAGUGUGACCCGAAAGGGCUCGCAAGUGCCGGAUUCU 60
TH31 1:accauUAAAGCCACUCGGUGGUUAAGUGUGACCCGAAAGGGCUCGCAAGUGCCGGAUUCU 60
PV33 1:accauU-AAGCCACUCGGUGGUUGAGUGUGACCCGAAAGGGCUCGCAAGUGCCGGAUUCU 59
PV34 1:accauU-AAGCCACUCGGUGGUUGAGUGUGACCCGAAAGGGCUCGCAAGUGCCGGAUUCU 59
TH35 1:accauUAAAGCCACUCGGUGGUUAAGUGUGACCCGAAAGGGCUCGCAAGUGCCGGAUUCU 60
PV41 1:accauUAAAGCCACUCGGUGGUUAAGUGUGACCCGAAAGGGCUCGCAAGUGCCGGAUUCU 60
P3 P4 P5 P5a P5b P5.1 P5.1a
PV1 61:GGCGCGACACUGUCAAAUUGCGGGGACUCCCUAAAGCUCCAGGGACCGCCCACUCCACGA 120
PV2 61:GGCGCGACACUGUCAAAUUGCGGGGACUCCCUAAAGCUCCAGGGACCGCCCACUCCACGA 120
PV3 61:GGCGCGACACUGUCAAAUUGCGGGGACUCCCUAAAGCUCCAGGGACCGCCCACUCCACGA 120
TH9 60:GGCGCGACACUGUCAAAUUGCGGGGACUCCCUAAAGCUCCAGAGACCGCCCCCUCCACGA 119
PV28 61:GGCGCGACACUGUCAAAUUGCGGGGACUCCCUAAAGCUCCAGGGACCGCCCUUUCCACGA 120
TH31 61:GGCGCGACACUGUCAAAUUGCGGGGACUCCCUAAAGCUCCAGGGACCGCCCUUUCCACGA 120
PV33 60:GGCGCGACACUGUCAAAUUGCGGGGACUCCCUAAAGCUCCAGGGACCGCCCACUCCACGA 119
PV34 60:GGCGCGACACUGUCAAAUUGCGGGGACUCCCUAAAGCUCCAGGGACCGCCCACUCCACGA 119
TH35 61:GGCGCGACACUGUCAAAUUGCGGGGACUCCCUAAAGCUCCAGGGACCGCCCUUUCCACGA 120
PV41 61:GGCGCGACACUGUCAAAUUGCGGGGACUCCCUAAAGCUCCAGGGACCGCCCACUCCACGA 120
P
P5.1a P5.1 P5.2 P5.2a P5.2a P5.2 P5b P5a P5c
PV1 121:AAGCGGAGGCACGGGCACCAGGUUAACGACCUCGGGUACGGUAAUACUUCUGGAGAUGUA 180
PV2 121:AAGCGGAGGCACGGGCACCAGGUUAACGACCUCGGGUACGGUAAUACUUCUGGAGAUGUA 180
PV3 121:AAGCGGAGGCACGGGCACCAGGUUAACGACCUCGGGUACGGUAAUACUUCUGGAGAUGUA 180
TH9 120:AAGCGGAGUCGAGGGCACCAGGUUAACGACCUCGGGCACGGUAAUACUUCUGGAGAUGCA 179
PV28 121:AAGUGGAAUCGAGGGCACCAGGUUAACGACCUCGGGUACGGUAAUACUUCUGGAGAUGCA 180
TH31 121:AAGUGGAAUCGAGGGCACCAGGUUAACGACCUCGGGUACGGUAAUACUUCUGGAGAUGCA 180
PV33 120:AAGCGGAGGCACGGGCACCAGGUUAACGACCUCGGGUACGGUAAUACUUCUGGAGAUGUA 179
PV34 120:AAGCGGAGGCACGGGCACCAGGUUAACGACCUCGGGUACGGUAAUACUUCUGGAGAUGUA 179
TH35 121:AAGUGGAAUCGAGGGCACCAGGUUAACGACCUCGGGUACGGUAAUACUUCUGGAGAUGCA 180
PV41 121:AAGCGGAGGCACGGGCACCAGGUUAACGACCUCGGGUACGGUAAUACUUCUGGAGAUGUA 180
P5c P5 P4 P6 P6a P6b P6b P6a P6 P7
PV1 181:ACAAUGGGUAAUCUGCAGCCAAGUCCUACGGGCGAUCGCCUACGGAUGCAGUUCACAGAC 240
PV2 181:ACAAUGGGUAAUCUGCAGCCAAGUCCUACGGGCGAUCGCCUACGGAUGCAGUUCACAGAC 240
PV3 181:ACAAUGGGUAAUCUGCAGCCAAGUCCUACGGGCGAUCGCCUACGGAUGCAGUUCACAGAC 240
TH9 180:ACAAUGGGUAAUCUGCAGCCAAGUCCUACGGGCGAUCGCCUACGGAUGCAGUUCACAGAC 239
PV28 181:ACAAUGGGUAAUCUGCAGCCAAGUCCUACGGGCGAUCGCCUACGGAUGCAGUUCACAGAC 240
TH31 181:ACAAUGGGUAAUCUGCAGCCAAGUCCUACGGGCGAUCGCCUACGGAUGCAGUUCACAGAC 240
PV33 180:ACAAUGGGUAAUCUGCAGCCAAGUCCUACGGGCGAUCGCCUACGGAUGCAGUUCACAGAC 239
PV34 180:ACAAUGGGUAAUCUGCAGCCAAGUCCUACGGGCGAUCGCCUACGGAUGCAGUUCACAGAC 239
TH35 181:ACAAUGGGUAAUCUGCAGCCAAGUCCUACGGGCGAUCGCCUACGGAUGCAGUUCACAGAC 240
PV41 181:ACAAUGGGUAAUCUGCAGCCAAGUCCUACGGGCGAUCGCCUACGGAUGCAGUUCACAGAC 240
Q
P3 P8 P8 P7 P9.0 P9 P9
PV1 241:UAGAUGGCAGUGGGCUCUCUCAGACGAGCUUAAGAUAUAGUCGGUCCCCUGGGGAGACCC 300
PV2 241:UAGAUGGCAGUGGGCUCUCUCAGACGAGCUUAAGAUAUAGUCGGUCCCCUGGGGAGACCC 300
PV3 241:UAGAUGGCAGUGGGCUCUCUCAGACGAGCUUAAGAUAUAGUCGGUCCCCUGGGGAGACCC 300
TH9 240:UAGAUGGCAGUGGGCUCUCACA---GAGCUUAAGAUAUAGUCGGUCCCCUGGGGAGACCC 296
PV28 241:UAGAUGGCAGUGGGCUCCCA-ACACGAGCUUAAGAUAUAGUCGGUCCCCUGGGGAGACCC 299
TH31 241:UAGAUGGCAGUGGGCUCCCA-ACACGAGCUUAAGAUAUAGUCGGUCCCCUGGGGAGACCC 299
PV33 240:UAGAUGGCAGUGGGCUCUCUCAGACGAGCUUAAGAUAUAGUCGGUCCCCUGGGGAGACCC 299
PV34 240:UAGAUGGCAGUGGGCUCUCUCAGACGAGCUUAAGAUAUAGUCGGUCCCCUGGGGAGACCC 299
TH35 241:UAGAUGGCAGUGGGCUCCCA-ACACGAGCUUAAGAUAUAGUCGGUCCCCUGGGGAGACCC 299
PV41 241:UAGAUGGCAGUGGGCUCUCUCAGACGAGCUUAAGAUAUAGUCGGUCCCCUGGGGAGACCC 300
R S
P9.1 P9.1a P9.1b P9.1c P9c1a P9.1b P9.1a
PV1 301:AGGCGCAUCGCUAAACUGGAGGUGAAGGAUUCUGCCCCCC--GGGCAGUCACCCCCAGCC 358
PV2 301:AGGCGCAUCGCUAAACUGGAGGUGAAGGAUUCUGCCCCCC--GGGCAGUCACCCCCAGCC 358
PV3 301:AGGCGCAUCGCUAAACUGGAGGUGAAGGAUUCUGCCCCCC--GGGCAGUCACCCCCAGCC 358
TH9 297:AGGCGCAUCGCUAAACUGGAGGUGAAGGAUUCUGCCCCCCCGGGGCAGUCACCCCCAGCC 356
PV28 300:AGGCGCAUCGCUAAACUGGAGGUGAAGAAUUCUGCCCCC---GGGCAGUCACCCCCAGCC 356
TH31 300:AGGCGCAUCGCUAAACUGGAGGUGAAGAAUUCUGCCCCC---GGGCAGUCACCCCCAGCC 356
PV33 300:AGGCGCAUCGCUAAACUGGAGGUGAAGGAUUCUGCCCCCC--GGGCAGUCACCCCCAGCC 357
PV34 300:AGGCGCAUCGCUAAACUGGAGGUGAAGGAUUCUGCCCCCC--GGGCAGUCACCCCCAGCC 357
TH35 300:AGGCGCAUCGCUAAACUGGAGGUGAAGAAUUCUGCCCCC---GGGCAGUCACCCCCAGCC 356
PV41 301:AGGCGCAUCGCUAAACUGGAGGUGAAGGAUUCUGCCCCCC--GGGCAGUCACCCCCAGCC 358
P9.1 P9.2 P9.2 P9.0 P10
PV1 359:GGAACGGUGCGGGCACCCGUGUGGUGCUGGAGUAAACGCuggu 401
PV2 359:GGAACGGUGCGGGCACCCGUGUGGUGCUGGAGCAAACGCuggu 401
PV3 359:GGAACGGUGCGGGCACCCGUGUGGUGCUGGAGUAAACGCuggu 401
TH9 357:GGAACGGUGCGGGCACCCGUGCGGUGCUGGAGUAAACGCuggu 399
PV28 357:GGAACGGUGCGGGCACCCCCGCGGUGCUGGAGCAAACGCuggu 399
TH31 357:GGAACGGUGCGGGCACCCCCGCGGUGCUGGAGCAAACGCuggu 399
PV33 358:GGAACGGUGCGGGCACCCGUGUGGUGCUGGAGUAAACGCuggu 400
PV34 358:GGAACGGUGCGGGCACCCGUGUGGUGCUGGAGUAAACGCuggu 400
TH35 357:GGAACGGUGCGGGCACCCCCGCGGUGCUGGAGCAAACGCuggu 399
PV41 359:GGAACGGUGCGGGCACCCGUGUGGUGCUGGAGUAAACGCuggu 401
