# Supplementary material for: Occurrence and characteristics of group 1 introns found at three different positions within the 28S ribosomal RNA gene of the dematiaceous Phialophora verrucosa: phylogenetic and secondary structural implications
Source: BMC Microbiol. 2011 May 8;11:94. doi: 10.1186/1471-2180-11-94 (PMC3112068; doi:10.1186/1471-2180-11-94)
Supplement: Additional file 4 — Alignment of intron-G used for the phylogenetic analysis and the modeling of secondary structure. The gaps were marked with dashes. The highly conserved (ribozymatic core) regions of the P, Q, R and S were marked with dotted lines. Boxed nucleotides participate in the pairing segments of P1-P10 of the secondary structure model. [file 1471-2180-11-94-S4.PDF]

```
P1                P1a          P1b        P1c                 P1c              P1b        P1a
PV1    1:augacuCAACUGGAACUACUACCGGGAGGUGACUUAAAG--GUCACCUAU--CCCUUUUG 56
PV33   1:augacuCAACUGGAACUACUACCGGGAGGUGACUUAAA---GUCACCUAU--CCCUUUUG 55
PV34   1:augacuCAACUGGAACUACUACCGGGAGGUGACUUAAA---GUCACCUAU--CCCUUUUG 55
PV3    1:augacuCAACCAGAACUACCAGGGGGACGGACUGACGGAGUCAUCUGUCCCCCCUUUG 60
         **********..******.*..***...****.*.. ****.**.* ***.****
       P1a  P1b  P1c   P1c  P1b  P1a  P10  P1   P2.0

P10     P1          P2  P2a        P2a    P2     P2.1                 P2.1
PV1   57:UAAGAGGGUCAUUAGAGCGGCGUAAAAGGCGACCGCUAGUGACCUGUUGGCUUCAACAGG 116
PV33  56:UAAGAGGGUCAUUAGAGCGGCGUAAAAGGCGACCGCUAGUGACCUGUUGGCUUCAGCAGG 115
PV34  56:UAAGAGGGUCAUUAGAGCGGCGUAAAAGGCGACCGCUAGUGACCUGUUGGCUUCAGCAGG 115
PV3   61:UGAGAGGGUCAUCAAAGCGGCGUAAAAGGCGACCGCUAGUGACCUGUUGGCUUCAACAGG 120
         *.**********.*.*********************************** ****

      P3        P4         P5       P5a      P5b     P5.1
PV1  117:UUGCAACAUCAUCAAAUUGCGGGAAGUUCCUAAAGCUCAAGCUACCAAGCGUCCGAUGAA 176
PV33 116:UUGCAACAUCAUCAAAUUGCGGGAAGUUCCUAAAGCUCAAGCUACCAAGCGUCUGAUGAA 175
PV34 116:UUGCAACAUCAUCAAAUUGCGGGAAGUUCCUAAAGCUCAAGCUACCAAGCGUCUGAUGAA 175
PV3  121:UUGCAACAUCAUCAAAUUGCGGGAAGUUCCUAAAGCUCAAGCUACCAAGCGUCCGGUGAA 180
         ********************************************************* *.****
                    P

    P5.1     P5.2  P5.2a   P5.2a   P5.2  P5b     P5a     P5c   P5c
PV1  177:AGUCGGGCGUGGCCGGGGUAAUGACCUAGGGUAUGGUAACAACGCUUGAGAUGCAACAAU 236
PV33 176:AGUCGGGCGUGGCCGGGGUAAUGACCUAGGGUACGGUAACAACGCUUGAGAUGCAACAAU 235
PV34 176:AGUCGGGCGUGGCCGGGGUAAUGACCUAGGGUACGGUAACAACGCUUGAGAUGCAACAAU 235
PV3  181:AGUCGGGCGUGGCCGGGGUAAUGACCUAGGGUACGGUAACAACGCUUGAGAUGUAACAAU 240
         ********************************.*******************.******

P5       P4    P6   P6a        P6b        P6b        P6a     P6
PV1  237:GGAUGAUCCGCAGCCAAGUCCUAAGGGCCCUUCUCGGGGGGCUACGGAUGCAGUUCAACG 296
PV33 236:GGAUGAUCCGCAGCCAAGUCCUAAGGGCCCUUCUCGGGGGGCUACGGAUGCAGUUCAACG 295
PV34 236:GGAUGAUCCGCAGCCAAGUCCUAAGGGCCCUUCUCGGGGGGCUACGGAUGCAGUUCAACG 295
PV3  241:GGAUAAUCCGCAGCCAAGUCCUAAGGCCCCCU-UCUCGGGGCUACGGAUGCAGUUCAACG 299
         ****.*************************.***.*.**..************************
          Q                                                   R

P7       P3      P8      P8a        P8b        P8b       P8a       P8
PV1  297:ACUAAAUGGUGGUGGGUGCACCUGGAGCACGCCCUUGUGGCGCACCUCCAGGUGGGCUUA 356
PV33 296:ACUAAAUGGUGGUGGGUGCACCUGGAGCACGCCCUUGUGGCGCACCUCCAGGUGGGCUUA 355
PV34 296:ACUAAAUGGUGGUGGGUGCACCUGGAGCACGCCCUUGUGGCGCACCUCCAGGUGGGCUUA 355
PV3  300:ACUAAAUGGUGAUGGGUGCACCUGGAGCACGCCCUUGCGGCGCACCUCCCGGUGGGCUUA 359
         ***********.*************************.***********.**********
                  P8a      P8b      P8c       P8c       P8b       P8a

         P7       P9.0           P9        P9.0  P9.0  P10
PV1  357:AGAUAUAGUCUGGCGUGGGCUGAAAGGUCCGCGGUAUAUGcucuu 401
PV33 356:AGAUAUAGUCUGGCGUGGGCUGAAAGGCCCGCGGUAUAUGcucuu 400
PV34 356:AGAUAUAGUCUGGCGUGGGCUGAAAGGCCCGCGGUAUAUGcucuu 400
PV3  360:AGAUAUAGUCUGGCGUGGACUGAAAGGUCCACGGUAUAUGcucuu 404
         ******************.******** **.**************
          S
```
